# The need to integrate spirituality into sleep medicine as a dimension of whole-person, person-centered care

**DOI:** 10.1007/s44470-026-00137-0

**Published:** 2026-08-04

**Authors:** Rupsha Singh, Christina M. Puchalski, Alexander Moreira-Almeida, Brian Merry, David H. Rosmarin, Harold G. Koenig, Chandra L. Jackson

**Affiliations:** 1 Epidemiology Branch, National Institute of Environmental Health Sciences, National Institutes of Health, Department of Health and Human Services,, 111 TW Alexander Drive, MD A3-05, Research Triangle Park, NC 27709 USA; 2 George Washington Institute for Spirituality and Health, George Washington University, Washington, DC USA; 3 Section on Spirituality and Mental Health, Brazilian Psychiatric Association (ABP), Rio de Janeiro, RJ Brazil; 4 NUPES – Research Center in Spirituality and Health, School of Medicine, Federal University of Juiz de Fora - UFJF, Juiz de Fora, MG Brazil; 5 Spiritual Fitness Program, United States Air Force, San Antonio, TX USA; 6 Spirituality and Mental Health Program, McLean Hospital, Belmont, MA USA; 7 Department of Psychiatry, Harvard Medical School, Boston, MA USA; 8 Department of Psychiatry and Behavioral Sciences, and Department of Medicine, Duke University Medical Center, Durham, NC USA; 9 Division of Intramural Research, National Institute on Minority Health and Health Disparities, National Institutes of Health, Department of Health and Human Services, Bethesda, MD USA

Sleep—an essential pillar of health—has historically been considered not only a biological phenomenon but also based on experiences interpreted through spiritual, moral, existential, and cultural frameworks worldwide [1–5]. Across societies, dreams, nightmares, sleep paralysis, and nocturnal visions have frequently been attributed to soul journeys, spirits, precognitions, moral conflict, supernatural attack, and ancestors [2, 4–6]. Yet, contemporary sleep medicine remains predominantly grounded in biomedical models despite growing recognition of more holistic frameworks. For instance, Whole Person Health—developed by the National Center for Complementary and Integrative Health—adopts a biopsychosocial-spiritual approach that recognizes physical, mental, social, and spiritual dimensions of health and wellbeing are interconnected and can collectively shape health outcomes relevant to sleep [7].

Religion and spirituality remain highly prevalent globally, with approximately 75.8% of the world’s population affiliated with a religious tradition [8]. Additionally, nearly one-quarter of the global population is religiously unaffiliated, yet many continue to identify as spiritual or endorse spiritual beliefs and practices despite lacking formal religious affiliation [9]. Sleep-related spiritual experiences are also common; 53% of US adults report dream or other experiences involving deceased loved ones [10], 70% of Brazilians report at least one precognitive dream [11], and out-of-body experiences—including those occurring during sleep or sleep–wake transitions—have been reported by up to 20% of the population [12]. Such experiences may become sources of distress and prompt healthcare seeking, particularly when individuals lack cultural, spiritual, or social frameworks through which to understand them [13].

Underscoring its clinical relevance, the American Psychiatric Association expanded—in 2025—the DSM-5-TR code Z65.8 from “Religious or Spiritual Problem” to “Moral, Religious, or Spiritual Problem,” recognizing moral and spiritual distress/conflict as a clinically significant foci warranting attention without pathologizing them as psychiatric disorders [14]. Such concerns are likely relevant to sleep medicine given evidence associating trauma exposure, moral injury, and spiritual distress with nightmares, hyperarousal, and insomnia symptoms [15].

Furthermore, religion and spirituality are increasingly recognized as determinants of sleep [16, 17]. For instance, some studies report associations between religious service attendance, positive religious coping, and spiritual well-being with better sleep quality and longer sleep duration, whereas religious struggle, spiritual distress, religious doubt, or guilt have been associated with poorer sleep outcomes [17]. Potential pathways through which religion and/or spirituality may influence sleep include enhanced coping resources, resilience to stress, reduced neural reactivity to negative emotional stimuli, and neurobiological correlates associated with lower risk of depression and improved emotional regulation [18]. Given their influence on coping strategies, illness interpretation, and medical decision making, spiritually-sensitive care should be more fully integrated into sleep medicine.

Spiritual care has been increasingly integrated into healthcare practice and professional training, reflecting growing recognition of spirituality as an important component of whole-person care. Brief evaluation of patients’ spiritual needs (known as a spiritual history) during the clinical encounter may help identify concerns relevant to sleep health and support appropriate follow-up, referral, and whole person sleep care [13]. Educational initiatives such as the Interprofessional Spiritual Care Education Curriculum (ISPEC©) provide clinicians with training and competencies to address patients’ spiritual needs in clinical settings [19, 20]. Importantly, incorporating spirituality into sleep medicine does not require clinicians to endorse religious beliefs nor engage in pastoral counseling. Rather, spiritually sensitive sleep care involves recognizing when existential, spiritual, or cultural frameworks meaningfully shape patients’ sleep experiences, distress, coping, and treatment engagement to better support clinical communication, therapeutic alliance, and person-centered, culturally responsive treatment planning. Some healthcare systems such as Veterans Affairs have integrated spiritual care through interdisciplinary teams including chaplains, providing support grounded in deep listening, presence, and person-centered care. Related initiatives such as military spiritual fitness programs further reflect growing institutional recognition of spirituality, meaning, values, and purpose as components of resilience, wellbeing, and readiness. Moreover, many patients wish to discuss spiritual concerns with clinicians [21]. Randomized clinical trials have also demonstrated the efficacy of integrating spiritual practices into cognitive-behavioral interventions, suggesting potential applications for behavioral sleep interventions [22].

Cross-cultural sleep research further illustrates that spiritual interpretations of sleep are not limited to specific populations but represent a common way that individuals make meaning of sleep experiences. Such beliefs may influence symptom interpretation, help-seeking behaviors, treatment engagement, and medical decision making. For example, qualitative research among Māori in Aotearoa, New Zealand, conceptualizes sleep as a spiritually significant state in which the *wairua*(spirit) awakens, journeys, learns, and provides guidance for waking life [2]. Likewise, in several African cultural traditions, dreams may be interpreted as sources of guidance, prophecy, or spiritual influence, shaping how sleep experiences and sleep disturbances are understood [3]. For example, among Yoruba communities in Nigeria, nightmares and dreams may be interpreted as manifestations of witchcraft [3]. Experiences such as sleep paralysis, sensed presence, or vivid dream encounters may, therefore, become pathologized/stigmatized when interpreted outside their cultural and spiritual context.

As medicine increasingly advances toward precision and person-centered care models, this serves as a call to action for greater attention to the influence of religion and spirituality on the practice of sleep medicine, in order to strengthen therapeutic alliance, improve culturally and spiritually responsive care, and advance more holistic approaches to sleep health.

## Data Availability

Not applicable.
